# Better Burning, Better Breathing: Improving Health with Cleaner Cook Stoves

**DOI:** 10.1289/ehp.118-a124

**Published:** 2010-03

**Authors:** Tina Adler

**Affiliations:** **Tina Adler** has written for *EHP* since 1993. She is a member of the National Association of Science Writers and the American Society of Journalists and Authors

Every morning and evening, millions of women in India spend an hour or two cooking their rice, dal, curry, and roti or other flat bread. Most will prepare their meals over a smoky, 3-stone open fire or a traditional clay or brick cook stove called a chulha. The stoves burn a mix of wood, hay, or cow dung that the women collect from around their homes or, at times, far from the safety of their villages. The old-fashioned chulhas cook slowly, imparting a delicious flavor to the food that many Indians love. But everyone—from the women cooking their meals to international health experts—knows the smoke from the fires has a dark side, literally and figuratively.

Users say the smoke burns their eyes and blackens their pots and kitchen walls. Climatologists such as Veerabhadran Ramanathan, a professor at the University of California, San Diego, say the black carbon (soot) from the smoke, which blankets the villages, contributes to anthropogenic climate change. Health experts report that smoke exposure increases the risk of numerous diseases. “Day in and day out, and for hours at a time, women and their small children breathe in amounts of smoke equivalent to consuming two packs of cigarettes per day,” the World Health Organization (WHO) reported in the 2006 report *Fuel for Life: Household Energy and Health*.

To date, the few nongovernmental organizations, companies, and development and public health agencies that have tried to replace these traditional stoves have met with only isolated successes in large-scale programs. Now India intends to launch one of the largest improved cook stove initiatives in the world, its government announced in December 2009. Although several other governments have stove replacement programs, India’s has a larger potential than most.

Clean-stove experts warn there is no one perfect stove or stove program that will solve India’s—or any other country’s—problem with smoke exposure. “Almost certainly, we need a range of options for different groups defined by geography, socioeconomic level, and culture,” says Nigel Bruce, a consultant to the WHO and senior lecturer at the University of Liverpool. “It is possible that a common technology could be at the core of these, but I would not assume that either.”

## Where There’s Smoke, There’s Disease

Researchers can now list the ingredients of the smoke flowing from open fires and old-fashioned cook stoves. Most—about 90%—is carbon monoxide (CO). The rest is a mix of volatile organic compounds, polyaromatic hydrocarbons, metals, and particulate matter including PM_10_ (which easily penetrates airways) and PM_2.5_ (the smaller fraction, which penetrates deep into the lungs). According to *Fuel for Life*, 24-hour levels of PM_10_ in homes that use solid fuels routinely reach 300–3,000 μg/m^3^ and may spike to 10,000 μg/m^3^ during cooking. By comparison, the WHO recommends no more than an annual mean of 20 μg/m^3^ and a 24-hour mean of 50 μg/m^3^ for PM_10_.

Breathing the smoke from traditional cook stoves and open fires may have caused 1.96 million premature deaths worldwide in 2004, according to WHO risk factor estimates accompanying the 2008 report *The Global Burden of Disease: 2004 Update*. The diseases and conditions associated with smoke exposure include pneumonia, chronic respiratory disease, heart disease, low birth weight, and probably tuberculosis and other diseases. Globally, an estimated 49% of deaths attributable to household use of solid fuel are due to pneumonia in children under age 5. In a meta-analysis of 24 studies in the May 2008 issue of the *Bulletin of the World Health Organization*, Bruce and colleagues reported that, despite “substantial” differences among the studies, “this analysis demonstrated sufficient consistency to conclude that risk of pneumonia in young children is increased by exposure to unprocessed solid fuels by a factor of 1.8.”

The data on illnesses related to cook stoves come primarily from observational studies; researchers have very little data from randomized controlled trials. To address this gap, a team led by Kirk Smith, director of the Global Health and Environment Program at the University of California, Berkeley, undertook a randomized cook stove trial in the rural highlands of San Marcos, Guatemala. Called RESPIRE (Randomized Exposure Study of Pollution Indoors and Respiratory Effects), the trial involved 534 families. Half the group received a cook stove with a chimney, and the other half continued to use their fires or stoves without a chimney. The researchers monitored smoke exposure via a passive diffusion CO tube attached to the mothers’ and children’s clothing. CO is a reliable surrogate for fine particles and is easier to measure over time than other pollutants, according to Smith.

The use of stoves with chimneys in Guatemala reduced CO levels in the kitchen by about 90% and children’s exposure by an average 50% over 48 hours, the researchers reported online 17 June 2009 ahead of print in the *Journal of Exposure Science & Environmental Epidemiology*. However, Bruce points out, children are exposed to smoke when they go outside and when they go to other homes, so their individual CO exposure levels don’t match levels in their own kitchen.

## Is Better Good Enough?

Data from studies such as RESPIRE will help answer an important question facing clean-stove advocates and public health experts: how much must concentrations of smoke in homes be reduced in order to improve families’ health? When tested in the field, few of the improved cook stoves used in India achieve more than a 50–60% reduction in indoor air pollution levels and a 50% reduction in fuel use, says Simon Bishop, policy and communications manager at the Shell Foundation, which promotes improved cook stoves as a primary solution to indoor air pollution.

Whereas manufacturer testing in the laboratory may indicate a stove is capable of producing far less CO and suspended PM_10_ compared with traditional cook stoves, field conditions—particularly the significant natural variations in operator behavior and the type, size, and moisture content of the fuel used—can lead to substantially lower performance than might be predicted from lab tests, according to Smith. There are advanced stoves but “we don’t think any of them are truly as advanced as they should be,” Smith says.

“The existing improved stoves have to go some way before they can meet a health-based standard, but they are much, much better than the traditional stoves we have now,” says Kalpana Balakrishnan, head of the Department of Environmental Health Engineering at Sri Ramachandra University in Chennai. Studies by Balakrishnan and colleagues suggest even existing cleaner cook stoves will contribute to an immediate improvement in children’s health. And for many populations, especially the poorest, it may be that current technologies are a reasonable intermediate step—just “not a policy end point,” says Bruce. He adds, “We have to be pragmatic.”

None of the existing stove technology was commercially available 3 years ago, and even better devices will be introduced in the next 3 years, points out Jacob Moss, a senior advisor at the U.S. Environmental Protection Agency (EPA) and director of the Partnership for Clean Indoor Air, an international collaboration comprising more than 340 partners. “If supply and distribution networks can be developed now, the newer technologies will be able to be substituted into them as they reach the market,” Moss says.

## A Stove in Every Kitchen?

In the early 1980s, India and China both began ambitious countrywide clean-stove programs. Today, China has about 150 million improved stoves in use from that time, and India has none, says Smith, who has studied both programs in depth. China used an innovative approach of encouraging local development of sophisticated stove designs and a central production and distribution system for key parts of the stove, says Smith. The Indian program failed for many reasons, including the fact that the distributed stoves made by local artisans produced too much smoke and were not durable. In addition, stove designs did not always consider the availability of reliable fuel.

But India is ready to try again with a whole new strategy. The new program, the National Biomass Cookstove Initiative, is intended to provide cleaner, more efficient biomass-fueled stoves to rural communities, the Indian Ministry of New and Renewable Energy announced in a 2 December 2009 press release. The new program will draw lessons from and build on the successes of the earlier program.

The government appears committed to independent monitoring and evaluation of the stoves and to focusing on vulnerable groups, Smith says. “This is seen as an entirely domestic program—no foreign aid or carbon financing is being sought,” he explains. Technical committees of public health, clean-stove, and marketing experts have met to develop best practice recommendations, set up a timetable for the program, establish standards for stove performance, and share strategies for marketing the stoves, among other tasks. The goal of the program is to sell 150 million stoves in 10 years, says Harish Anchan, general manager of stove manufacturer Envirofit India. The government is expected to release more information on the program in March 2010.

India decided to launch another program in part because “stove technology has improved considerably in the past few years,” the government announcement notes. The government will remain open to using better stoves as they become available—advances in stove technology “are still possible and, indeed essential,” the government release states. As part of the new initiative, a multimillion-dollar “innovation prize” will be awarded to encourage the development of improved cook stoves, says Smith.

The initiative will also include an accreditation component. Knowing the health effects of different concentrations of smoke will give international agencies and governments the data necessary to establish “clean cook stove” standards that manufacturers must reach to be accredited. This ensures that customers who purchase an accredited “clean cook stove” can expect certain improvements in fuel use and reductions in indoor air pollution levels, says Anchan.

## Making It Work

The initiative is good news to stove experts such as Douglas Barnes, coauthor of the forthcoming book *Cleaner Hearths, Better Homes—Improved Stoves for India and the Developing World*. But Barnes also warns against launching a heavily subsidized program without the budget to back it up long-term. “I am actually in favor of subsidies,” he says, “just not ones that have a negative impact on programs.” Heavily subsidized programs often are not self-sustaining, because when the budget for the program dries up, so does the program.

Subsidies can prevent the development of markets in general, and improved cook stoves are no exception, Barnes says. He predicts that a subsidy for the stove itself of more than 20–25% would prevent the market from taking off. People who use biomass-fueled stoves are generally poor, and the initial cost of purchasing a new stove is a major problem for them. The involvement of microfinance organizations—which loan money to people too poor to borrow from a regular bank—may be one solution to this problem. However, Barnes says the current effort by the government of India appears to steer away from past high-subsidy approaches, so if the new effort is done properly, it could give a tremendous boost to cook stove programs worldwide.

The Indian government isn’t alone in its effort to expand Indians’ access to cleaner biomass cook stoves. International donors such as the Shell Foundation are increasing their support of cook stove programs that show the potential for economic sustainability and scalability. Corporations such as Royal Philips Electronics, First Energy (formerly a BP company), and Bosch-Siemens are developing cleaner cook stoves that can be customized for cooking needs around the world. Companies that can manufacture stoves include Envirofit, StoveTec, First Energy, WorldStove, and HELPS International.

The Shell Foundation has invested $3.5 million in Envirofit to support its program to sell 5–7 million stoves in 7 states in India in the next 5 years and is investing several million dollars in a public awareness campaign. The Shell Foundation and Envirofit are also in “close talks with the Indian government to see [how] we would work with them,” says Bishop. “As part of our national [indoor air pollution] awareness-raising campaign, we planned on lobbying the government to get indoor air pollution higher up on the agenda, so the government has almost jumped ahead of us—and it’s great that they have.”

## Selling the Idea, Finding the Funds, Providing the Support

The main objective of Indian consumers who purchase an improved cook stove is often quite different from the Indian government’s reason for promoting stoves. The government wants to improve the health of rural Indians. “But people buy [stoves] more for aspirational reasons,” says Mahesh Yagnaraman, CEO and managing director of First Energy. The energy-efficient stoves burn less, or burn different fuels, so women and children spend less time gathering solid fuel. As part of its original sales campaign, First Energy had doctors test the breathing capacity of interested customers to demonstrate how the old stoves had harmed their lungs. But instead of wanting a new stove, the prospective customers “just wanted medicine,” Yagnaraman says.

To sell stoves, you have to explain how the stoves save time and fuel and don’t dirty the kitchen with soot, says Anchan. Selling improved stoves is “very difficult,” he says. Consumers know the smoke irritates their eyes and makes them cough, but they don’t always comprehend the long-term impact of breathing high levels of smoke.

Customers “don’t trust any of these [stove] solutions very easily,” says Yagnaraman. No stove is entirely reliable, so some families will keep more than one type of stove in their homes, he says. Usage studies show that families cook at most about 70% of their meals on their improved cook stove, says Smith. In their studies, he and his colleagues attach microchip usage monitors to stoves instead of relying on self-reports from the family members. “If you ask people, they say, ‘Oh yes, we love it, we use it all the time,’” Smith says.

Significant customer support is key to any cook stove program’s success, experts say. “You can’t drop a stove into a household and walk away,” notes Rita Colwell, a public health expert at the University of Maryland at College Park and the Johns Hopkins University Bloomberg School of Public Health. Envirofit provides that hands-on care, Anchan says. In addition to training customers how to use the stove and providing them with a manual, Envirofit calls customers to see how the stove is working for them.

One of the biggest hurdles for stove engineers and manufacturers is producing a stove that is both affordable and well made. Companies know how to make a stove that has very low emissions, is sturdy, relatively easy to use, and reliable but not necessarily at a price that consumers can afford, notes Smith. So far most companies are marketing only to consumers who can afford to pay about US$20 for a stove, which excludes the very poor. But Envirofit plans to launch a new model that customers can purchase through monthly payments to a microfinance company, says Anchan. The user will pay about US$1 a month for around a year, he says. Another payment option that stove companies, including Philips, may pursue is to seek carbon credits for cleaner stoves.

## Next on the Agenda

Although smoke from cook stoves is clearly a significant public health issue, another one is larger: malnutrition. “We’ve just started to look at programs that are seeking to integrate household environmental interventions for household energy, water, and sanitation with nutrition,” says Bruce.

It’s unclear yet whether these integrated programs are more successful if run together or separately, Bruce says. There’s plenty of opportunity to find out: Experts estimate 600–800 million homes worldwide need improved cook stoves, and according to the United Nations Food and Agriculture Organization, more than 1 billion people were undernourished in 2009.

## Figures and Tables

**Figure:**
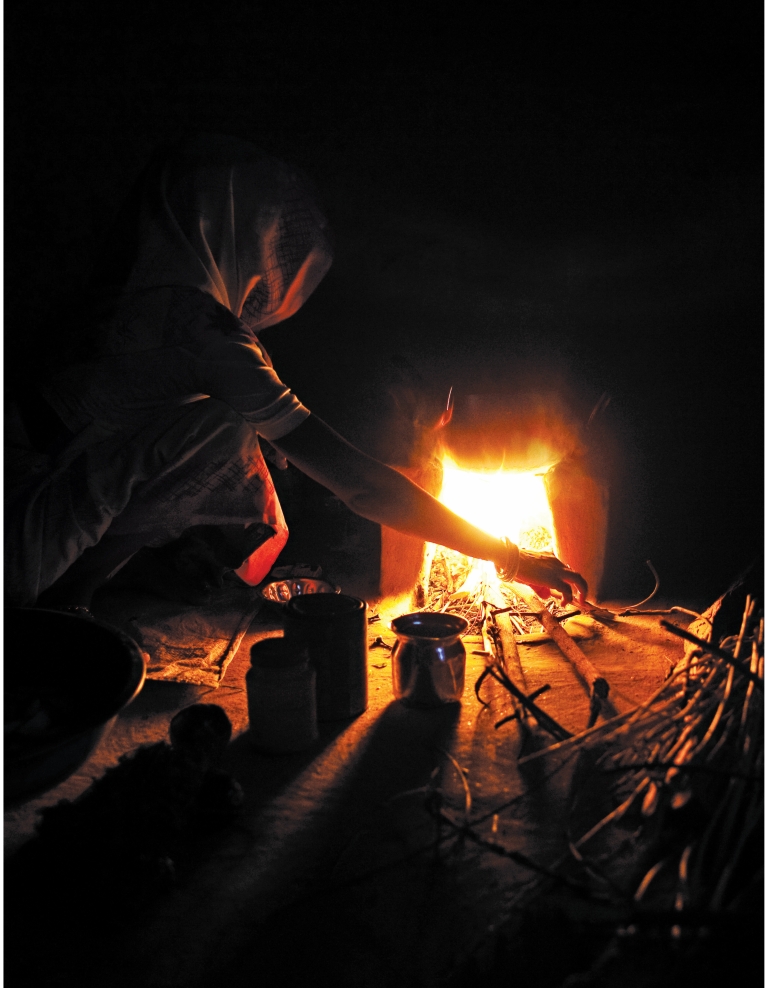


**Figure:**
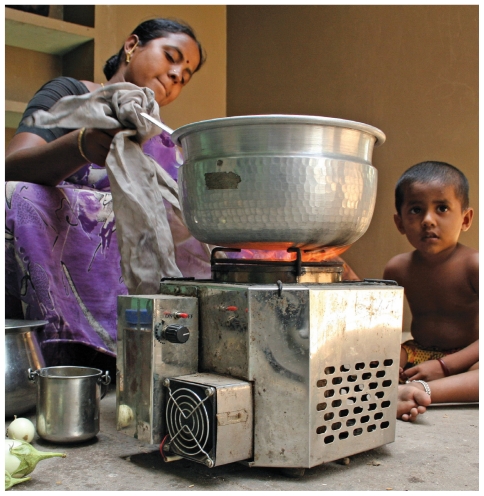
Opposite: Munnidevi Chhtrapalsingh cooks with fire inside her home in the village of Kolma, Uttar Pradesh, India. Kolma is participating in Project Surya (the Sanskrit word for “sun”), which aims to reduce atmospheric concentrations of black carbon by deploying cleaner-burning, energy-efficient cook stoves in homes such as this. This page: A family in Aviyur village, Tamil Nadu, cooks with a cost-effective stove that burns pellets made of agricultural waste.

**Figure:**
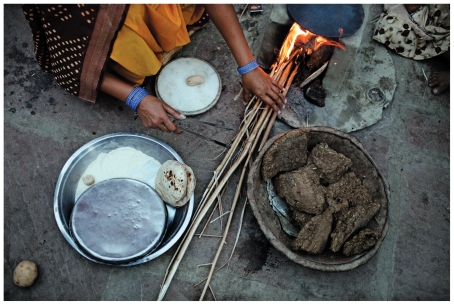
Siyaram Sikarwar cooks with a fire made from wood and cakes of cow dung at her home in Kolma, Uttar Pradesh. Around the world, women and children can spend up to several hours each day collecting these and other solid fuels to fire traditional cook stoves. The WHO estimates that breathing the smoke from traditional cook stoves and open fires caused nearly 2 million premature deaths worldwide in 2004, the most recent year for which there are data.

**Figure:**
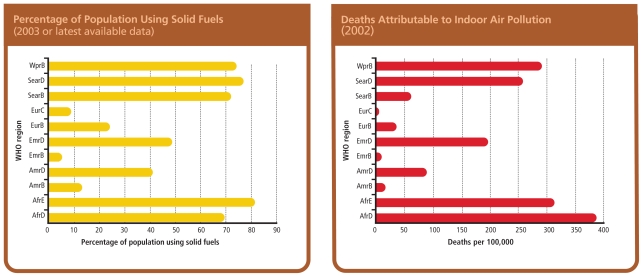
**Widespread Solid Fuel Use Translates into Respiratory Deaths** **Region abbreviations: Africa (Afr), the Americas (Amr), Eastern Mediterranean (Emr), Europe (Eur), Southeast Asia (Sear), and Western Pacific (Wpr). The WHO also differentiates among the following mortality strata: very low child/very low adult (A), low child/low adult (B), low child/high adult (C), high child/high adult (D), and high child/very high adult (E).** Source: WHO. 2006. Fuel for life: household energy and health. Geneva, Switzerland: World Health Organization; p. 13.

**Figure:**
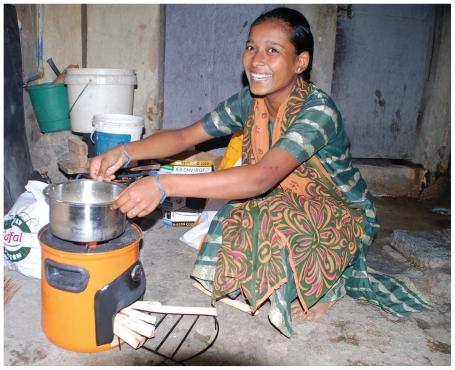
An estimated 600–800 million homes would benefit from a cleaner cook stove like this Envirofit G-3300 model.

**Figure:**
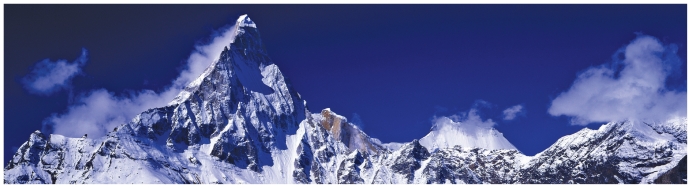
**As a result of incomplete combustion**, traditional cook stoves and open fires produce black carbon particles that sully kitchen walls and pots. These particles also reach the snow-covered Himalayas, darkening the once-white, reflective peaks and enhancing heat absorption, Ramanathan and colleagues reported in the 2 August 2007 issue of Nature. Black carbon from cook stoves and other sources such as burning of diesel fuel is accelerating ice melt on the Himalayas, William Lau and colleagues at the National Aeronautics and Space Administration reported in December 2009 at a meeting of the American Geophysical Union. Biomass cooking causes about two-thirds of black carbon emissions in South Asia, says Ramanathan. Last year he and his colleagues began the pilot phase of Project Surya, a study in India that seeks to measure reductions in atmospheric black carbon, methane, and ozone when families switch to cleaner stoves and lights. “In the Himalayas, the role of black carbon could be as high as fifty percent of the total glacial retreat we’ve seen so far,” he says.

**Figure:**
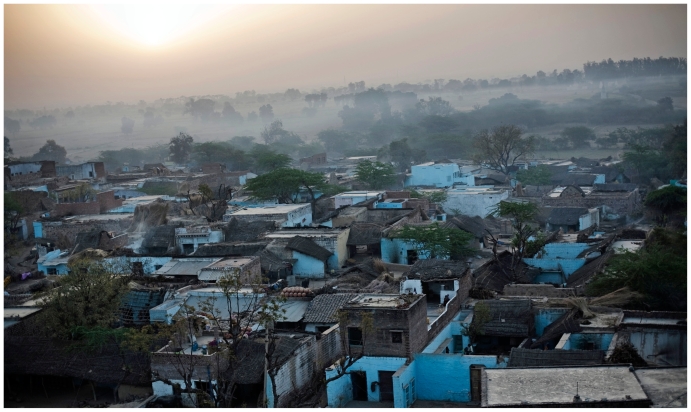
Smoke from morning fires hangs over the village of Pipal Kheda, Madhya Pradesh.

